# Effects of omeprazole and AlOH/MgOH on riociguat absorption

**DOI:** 10.1186/2050-6511-14-S1-P6

**Published:** 2013-08-29

**Authors:** Corina Becker, Reiner Frey, Sigrun Unger, Ulrike Artmeier-Brandt, Gerrit Weimann, Wolfgang Mueck

**Affiliations:** 1 Clinical Pharmacology, Bayer HealthCare Pharmaceuticals, Wuppertal, Germany; 2 Global Biostatistics, Bayer HealthCare Pharmaceuticals, Wuppertal, Germany

## Background

Riociguat, a soluble guanylate cyclase stimulator, is currently under investigation for the treatment of pulmonary hypertension. The present studies investigated the influence of omeprazole and AlOH/MgOH on riociguat absorption and bioavailability.

## Methods

The pharmacokinetics of oral, single-dose, immediate-release riociguat 2.5 mg were characterized in two open-label, randomized, crossover studies in healthy males. In the first study, subjects pretreated for 4 days with once-daily omeprazole 40 mg received co-treatment with omeprazole + riociguat or riociguat alone (no pretreatment) on Day 5 (n=12). In the second study, subjects received co-treatment with 10 mL AlOH/MgOH + riociguat or riociguat alone (n=12). Pharmacokinetic characteristics were analyzed assuming log-normally distributed data. Safety and tolerability were also assessed.

## Results

**Figure 1:**
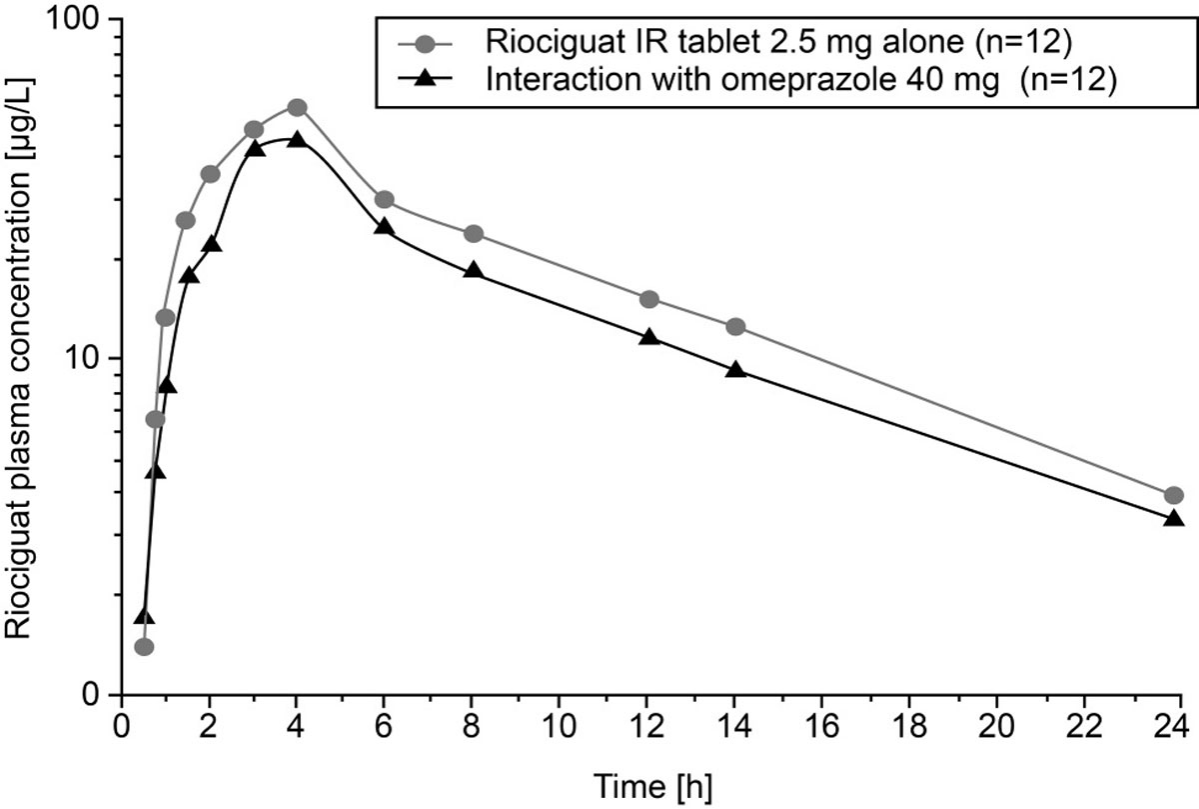
Riociguat plasma concentrations after a single oral dose of riociguat 2.5 mg with and without pre- and co-treatment with once-daily omeprazole 40 mg (geometric means, semilogarithmic scale; all subjects valid for pharmacokinetics; n=12). IR, immediate release.

**Figure 2:**
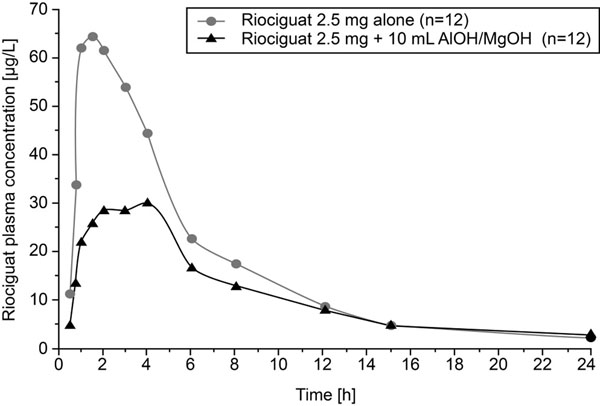
Riociguat plasma concentrations after a single oral dose of riociguat 2.5 mg with and without co-administration of 10 mL AlOH/MgOH (geometric means, semilogarithmic scale; all subjects valid for pharmacokinetics; n=12).

Riociguat bioavailability was decreased by pre- and co-treatment with omeprazole, with a mean decrease in C_max_ of 35% and a mean decrease in AUC of 26% (Table 1; Figure 1). Co-treatment with 10 mL AlOH/MgOH resulted in a mean decrease in C_max_ of 56% and a mean decrease in AUC of 34% (Table 1; Figure 2). In the riociguat/omeprazole study, adverse events (AEs) were reported in 4 (33%) subjects receiving riociguat alone and in 5 (42%) subjects receiving riociguat + omeprazole, with no AEs reported during the omeprazole pretreatment phase. The most commonly reported AEs were headache (9 events in 8 subjects; 5 drug-related events) and flushing (3 events in 2 subjects; all drug-related). In the riociguat/AlOH/MgOH study, AEs were reported in 9 (75%) subjects receiving riociguat alone and in 8 (67%) subjects receiving riociguat + AlOH/MgOH. The most commonly reported AEs were headache (12 events in 7 subjects; all drug-related), rhinitis (3 events in 3 subjects; no drug-related events), nasal congestion (3 events in 2 subjects; 2 drug-related events), and upper abdominal pain (3 events in 2 subjects; no drug-related events). No serious AEs were reported in either study and all AEs resolved by the end of the observation period.

**Table 1:** Riociguat pharmacokinetic parameters (geometric means and coefficients of variation)

	Riociguat/omeprazole study				Riociguat/AlOH/MgOH study			
	
Parameter^a^	Riociguat 2.5 mg (n=12)		Riociguat 2.5 mg + omeprazole (n=12)		Riociguat 2.5 mg (n=12)		Riociguat 2.5 mg + AlOH/MgOH (n=12)	
	
	GM	%CV	GM	%CV	GM	%CV	GM	%CV
AUC (µg⋅h/L)	587.9	71.9	432.8	79.8	465.9	68.2	309.6	87.2
C_max_ (µg/L)	73.8	26.6	48.1	33.8	80.8	38.4	35.5	57.1
t_max_ (h)	3.0	–	3.0	–	1.0	–	2.5	–
t_1/2_ (h)	7.9	46.4	9.0	25.4	5.9	44.4	8.6	53.8
CL/f	4.3	71.9	5.8	79.8	5.4	68.2	8.1	87.2

AUC, area under plasma concentration–time curve; CL/f, total riociguat clearance from plasma; C_max_, maximum riociguat plasma concentration; CV, coefficient of variation; GM, geometric mean; t_1/2_, elimination half-life; t_max_, time to reach C_max_.

^a^Data are mean except t_max_, which is median.

## Conclusion

Treatment with riociguat, with or without omeprazole or AlOH/MgOH, was well tolerated, with a good safety profile. The results confirm the lower bioavailability of riociguat in neutral versus acidic medium as expected from in vitro data. For co-medication of antacids like AlOH/MgOH, staggered intake between riociguat and antacid is practically possible and may be advisable. A general dose adaptation for patients with co-medication acting on gastric acidity, beyond the dose titration concept for riociguat, is not recommended.

